# Transcriptome Analysis Reveals miR-302a-3p Affects Granulosa Cell Proliferation by Targeting *DRD1* in Chickens

**DOI:** 10.3389/fgene.2022.832762

**Published:** 2022-03-30

**Authors:** Yufang Liu, Zuyang Zhou, Hui Zhang, Haiyin Han, Junqi Yang, Wenting Li, Kejun Wang

**Affiliations:** ^1^ College of Animal Sciences and Biotechnology, Henan Agricultural University, Zhengzhou, China; ^2^ College of Life Sciences and Food Engineering, Hebei University of Engineering, Handan, China

**Keywords:** DRD1, GC proliferation, Taihang chicken, small yellow follicles, miR-302a-3p

## Abstract

Egg production is an important economic trait in laying chickens as higher yields bring higher profits. Small yellow follicle (SYFL) development is a key determinant of chicken reproductive performance; however, the majority of SYFLs are not selected during the process of chicken reproduction and thus, atresia occurs. Although there have been numerous omic studies focused on egg production, the molecular mechanisms involved are still not well-understood. In this study, we used high-throughput technology to analyze the differences between the SYFL mRNA transcriptomes of high– (H) and low–egg-yielding (L) Taihang layer hens, with the aim of identifying the potential candidate genes involved in controlling the rate of egg production. We constructed six cDNA libraries, three from H and three from L Taihang hens and then performed high-throughput sequencing. Comparison of the H and L groups showed 415 differentially expressed genes (DEGs). In the high-yield group, 226 were upregulated and 189 were downregulated. Differentially enriched biological functions and processes were identified using Gene Ontology (GO) and Kyoto Encyclopedia of Genes and Genomes (KEGG) database analysis. Ten of the candidate DEGs we identified (*DRD1*, *MC5R*, *PCK1*, *CTSA*, *TGFBR3*, *AGO4*, *SLIT2*, *RGS1*, *SCNN1B*, and *ZP3*) have been identified in previous studies as being involved in the development of small yellow follicles. *DRD1* was significantly enriched in the gap junction pathway, which is an important pathway in chicken granulosa cells (GCs) to pass nutrition to an oocyte. Homology analysis showed that *DRD1* was highly conserved in numerous species, indicating that it may be a productive target for improving egg production. Evidence from bioinformatics analysis revealed that gga-miR-302a-3p putatively targets the 3′UTR region of *DRD1*. We then identified the functions of gga-miR-302a-3p in follicular granulosa cell proliferation by targeting *DRD1*. RT-qPCR analysis showed that *DRD1* and miR-302a-3p expression were inversely related in the SYLs of high and low egg-yielding chickens. Luciferase assays showed that miR-302a-3p targets the 3′UTR of *DRD1*, and overexpression of miR-302a-3p significantly inhibits the expression of *DRD1* in chicken GCs (p < 0.01). Functional experiments revealed that by targeting *DRD1*, miR-302a-3p acts as an inhibitor of GC proliferation. Taken together, we concluded that miR-302a-3p affects chicken GC proliferation by targeting *DRD1*. Our data expanded the knowledge base of genes whose functions are important in egg production and the molecular mechanisms of high-yield egg production in chicken small yellow follicles.

## Introduction

Follicle development, a key factor in determining reproductive performance, is influenced by physiology and the environment. The characteristics of follicle maturation in hens are like those in mammals, making them a useful model for studying follicle development in general (Bahr, 1991). The process of ovulation occurs through the recruitment of primordial follicles followed by primary follicles, small white follicles, large white follicles, small yellow follicles (SYFLs), and hierarchal follicles that progress toward maturity (Johnson, 2012). During the peak laying period of hens, ovarian hierarchal follicles develop from a pool of SYFLs each day (Hocking, 2009). In the process of hen egg production, the molecular and cellular mechanisms by which the pre-hierarchical follicles are selected to enter the preovulatory stage are well-known (Fortune et al., 2004; Johnson and Lee, 2016). It is estimated that there are approximately 12,000 oocytes in a sexually mature hen; however, only a few hundred of them are selected to mature and reach the ovulation stage (Onagbesan et al., 2009). Most SYFLs are not selected and undergo atresia (Tilly et al., 1991; Johnson et al., 1996). The SYFs continue to develop into the eight largest orderly arranged pre-ovulatory follicles, F8, F7, F6, F5, F4, F3, F2, F1 and proceed to ovulation (Huang et al., 2008). The development of SYFLs is crucial for ovulation in poultry (Dong et al., 2014).

Follicle development is a complex process, with numerous genes and pathways involved in their proliferation and differentiation (Woods and Johnson, 2005; Johnson et al., 2008). Dopamine receptor D1 (*DRD1*) is a type of D1-like receptor. In avians, dopamine participates in stimulating and inhibiting the secretion of prolactin (PRL) and plays a key role in the onset and maintenance of incubation behavior (Sharp et al., 1988; March et al., 1994; Youngren et al., 1995). Tempfli et al. found that *DRD1* promotes ovarian development by PRL and induces SYF differentiation in hens. Xu et al. reported that polymorphisms in *DRD1* affect the egg- laying performance of hens and that its haplotypes are significantly associated with some egg production traits in chickens (Xu et al., 2010; Tempfli et al., 2015). Wang et al. reported that *DRD1* may be a target gene for improving the characteristics of duck reproduction (Wang et al., 2012). Schnell et al. and Chaiseha et al*.* found that *DRD1* is expressed broadly in the hypothalamus and pituitary of turkeys and that its expression is related to reproductive function (Schnell and You, 1999; Chaiseha et al., 2003). In mammals, *DRD1* is expressed in many tissues, including the hypothalamus and thalamus (Fremeau et al., 1991; Jackson and Westlind-Danielsson, 1994), though not in the cerebellum, hippocampus, mesencephalon, or pituitary tissues (Monsma et al., 1990; Niznik and Van Tol, 1992; Vallone et al., 2000). The distribution of *DRD1* in the avian forebrain is largely the same as in mammals (Schnabel et al., 1997). Put together, these studies have revealed that *DRD1* is likely involved in regulating reproduction in birds.

The Taihang chicken, which produces high-quality eggs, is a breed native to Hebei Province in China. In this study, we used RNA-seq to investigate the molecular mechanisms of ovary follicle growth in high– and low–egg-yielding Taihang chickens. Differentially expressed genes were identified and then further examined to elucidate the potential functions and mode of regulation. We identified the *DRD1* as an important player in ovary follicle growth and miR-302a-3p as being potentially associated with follicle growth. These results provided a better understanding of chicken SYFL development and offered a target for subsequent investigation into improving the SYFL selected ratios.

## Materials and Methods

### Animals and Tissue Preparation

Four hundred Taihang hens raised at the Taihang Chicken Industry Co., Ltd. poultry breeding farm in Hebei Province were used in this study. At 33 weeks of age, three high-yield (high egg production, H) and three low-yield (low egg production, L) hens were selected from the same batch of laying hens. The laying rate in the H group was 68.33 ± 0.40%, and in the L group, it was 48.12 ± 0.40% (*p* < 0.05). The chickens had free access to food and water throughout the experiment and 12 h of natural light with temperatures between 17 and 25°C. The egg number was recorded daily at 16:00. The chickens were slaughtered by exsanguination, and the SYFLs were immediately harvested from the ovaries and stored in liquid nitrogen.

### RNA Isolation, Library Construction, and RNA-Seq

In accordance with the manufacturer’s instructions, total RNA was isolated from the six samples using TRIzol reagent (Life Technologies, Carlsbad, CA). The integrity of the isolated RNA (degradation and contamination) was determined by 1% agarose gels. RNA purity and concentration were determined by spectrophotometry using a NanoDrop ND-2000 (Implen, Westlake Village, CA). The RNA integrity number (RIN) of the samples ranged from 8.0 to 9.2; an RIN greater than 8.0 was considered acceptable for RNA-seq.

3 μg of the total RNA from each sample was used as the input material for library construction. Six libraries were generated using the NEB Next Ultra RNA Library Prep Kit for Illumina (New England Biolabs, Ipswich, MA) according to the manufacturer’s instruction. We added index codes to each sample for later identification. An Agilent Bioanalyzer 2100 system was used to determine the insert size of the libraries. The index-coded samples were clustered using the cBot Cluster Generation System and the TruSeq PE Cluster Kit v3-cBot-HS (Illumina, San Diego, CA), following the manufacturer’s instructions. The libraries were sequenced using an Illumina Hiseq X Ten platform to generate paired-end reads 150 bp in length (BGI Genomics Co., Ltd.).

### Raw Data Processing and Sequence Alignment

Raw reads were filtered using Trimmomatic (v0.36) (Bolger et al., 2014). In this step, the reads were removed from the data set if they included adapters, poly-N, or were of low quality. The clean reads were mapped to the *Gallus gallus* (v6.0) genome using HISAT2 (ver.2.2.1) with default parameters (Kim et al., 2015). Cufflinks were used to predict novel transcripts by comparing reconstructed transcripts with known transcripts (ver. 2.1.1) (Trapnell et al., 2012).

### Analysis of Differentially Expressed Genes

The reads mapped to each gene were counted by HiSeq (ver. 0.6.1). Gene expression levels were normalized by the Fragments Per Kilobase of transcript per Million map reads (FPKM) method (Trapnell et al., 2010). DESeq2 was used to analyze the differentially expressed genes from high– and low–egg-yielding chicken SYFLs (Anders and Huber, 2010). Genes with adjusted *p* ≤ 0.05 and |log2 (fold change) | ≥ 1 were classified as differentially expressed genes (DEGs).

### Confirmation of Differentially Expressed Genes

To validate the RNA-seq data, the expression levels of eight randomly selected DEGs were determined by RT-qPCR. Using a Fast First-Strand cDNA Synthesis kit (Tiangen, Beijing, China), RNA was reverse-transcribed onto cDNA. The primers used in the RT-qPCR were designed by Primer Premier 5 software (Premier Biosoft, Palo Alto, CA) and are shown in Supplementary Table S1. Each PCR reaction consisted of 1 μL of the cDNA template, 0.5 μL of forward and reverse primer (10 μmol), 10 μL of SYBR Green Master Mix (Tiangen, Beijing, China), and 8 μL of RNAase-free water. The amplification conditions were as follows: denaturation at 95°C for 5 min and then 40 cycles of amplification (95°C for 10 s and 60°C for 30 s). After amplification, melting curve analysis was performed by heating the samples to 95°C for 15 s, then cooling to 60°C for 1 min, followed by heating to 95°C at a rate of 0.3°C/s. Each sample was tested in triplicate. The expression level of the genes was calculated by the 2^*−ΔΔCt*^ method using *GAPDH* as a reference control (Livak and Schmittgen, 2001).

### DEG Functional Annotation Using GO and KEGG Enrichment Analyses

Gene Ontology (GO) enrichment analysis of the DEGs was carried out using the GOseq R package (ver. 2.12), with correction for gene length bias. Wallenius’ noncentral hypergeometric distribution was used to calculate *p*-values. GO terms with a corrected *p* value <0.05 were considered significantly enriched (Young et al., 2010). KOBAS (2.0) (Mao et al., 2005) was used to test for statistically significant enrichment of DEGs in the Kyoto Encyclopedia of Genes and Genomes (KEGG) pathway database.

### Prediction of miRNAs Targeting DRD1

The miRNA-targeting *DRD1* gene was predicted using TargetScan (http://www.targetscan.org/) and miRanda (http://www.miranda.org). miRNA–target relationships were considered significant at *p* < 0.05. To predict the conserved function of the *DRD1* gene, the sequencing results were subjected to a BLAST search (NCBI, http://blast.ncbi.nlm.nih.gov/Blast.cgi) to retrieve gene sequences homologous to *DRD1*. Multiple amino acid sequences were compared using DNAMAN software, and a phylogenetic tree was constructed using the Neighbor-Joining method in MEGA7 software (Kumar et al., 2016).

### Functional Assays

#### Vector Construction

Two recombinant psiCHECK2 vectors containing the predicted miR-302a-3p target site in the 3′- UTR sequence of *DRD1* were constructed using *XhoI* and *NotI* (Takara, Dalian, China). Wild and mutant-type recombinants are referred to as *DRD1*-3′UTR-WT and *DRD1*-3′UTR-MUT, respectively. The sequences of the wild and mutant- types were synthesized, and the sequences are shown in Supplementary Table S2. The miR-302a-3p sequence (CCA​CCA​CUU​AAA​CGU​GGA​UGU​ACU​UGC​UUU​GAA​ACU​AAA​GAA​GUA​AGU​GCU​UCC​AUG​UUU​UGG​UGA​UGG), was synthesized, digested with *BamHI* and *EcoRI*, and then inserted into pHBLV-U6-MCS-CMV-ZsGreen-PGK-PURO to obtain the LV021-miR-302a-3p. An empty vector served as a negative control (NC).

#### Cell Culture and Transfection

Following the methods described by Sharma et al., chicken primary granulosa cells (GCs) were isolated from SYFLs of ovarian tissues (Sharma and Sharma, 2020). The cells were seeded in 6-mm plates at a density of 10^5^ cells and maintained with a complete medium (DMEM/F12 (1:1), 10% FBS, and 1% penicillin/streptomycin) as described by Yang et al. (2017). HEK293T cells, obtained from our laboratory stock were seeded in 6-mm plates at a density of 10^5^ cells and maintained with a complete medium (DMEM/F12 (1:1), 10% FBS, and 1% penicillin/streptomycin).

#### Luciferase Assays

To validate the miRNA targets, 293T cells were cultured into 24-well plates and cotransfected with 200 ng *DRD1*-3′UTR-wt or *DRD1*-3′UTR-mut and 10 µL of miR-302a-3p, miR-302a-3p mimic, or negative control (NC) using Lipofectamine 2000 (Invitrogen). After 48 h, the cells were collected to measure the luciferase activity using a Dual-Luciferase Reporter Assay System Kit (Promega, WI, United States). All the experiments were performed in triplicates.

#### Cell Counting Kit-8 Assay

GCs were aliquoted into 96-well plates with approximately 100 μL of the cell suspension (about 10^3^−10^4^ cells) per well in three replicates. The cells were incubated for 2–4 h at 37°C after cell apposition for the CCK-8 assay. A Cell Counting Kit-8 (Beyotime, Beijing, China) was used to detect the GC proliferation following the manufacturer’s protocol. Absorbance at 450 nm was read at 0, 6, 12, 24, 48, and 72 h after the addition of CCK-8 solution.

### Statistical Analysis

Relative gene expression obtained from the RT-qPCR data was calculated using the 2^*−∆∆Ct*^ method (Stocks et al., 2012). All data were expressed as the mean ± SE (standard error) with at least three independent replicates and visualized using the “ggplot2” package in R (version 3.2.2, the University of Auckland, Auckland, New Zealand) and GraphPad Prism 7 software (San Diego, CA, United States). Student’s *t*-test was used to evaluate significant differences between the H and L groups. All data are shown as means ± standard error. *P* values <0.05 were defined as significant, and *p* values <0.01 were highly significant.

## Results

### RNA-Sequencing Data

**FIGURE 1:**
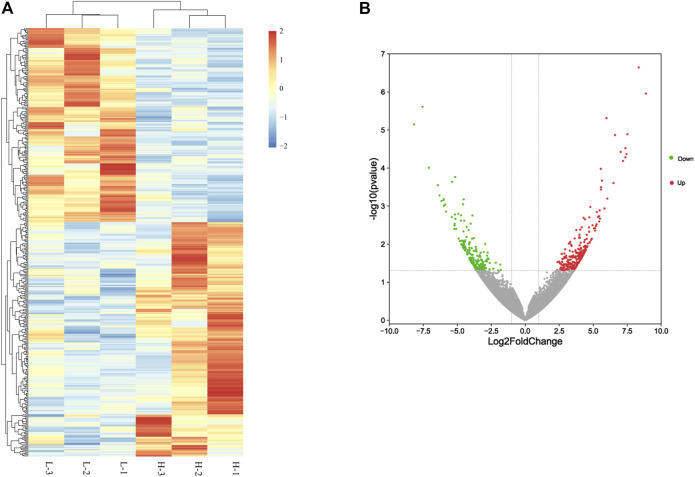
Differentially expressed genes between high– and low–egg-yielding groups of Taihang chicken. **(A)** Hierarchical clusters of differentially expressed genes. H represents the high–egg-producing group and L represents the low–egg-producing group. **(B)** Volcano plot of differentially expressed genes in chicken small yellow follicles. Red dots represent upregulated genes and green dots represent downregulated genes.

A summary of the sequencing data (Supplementary Table S3) shows that these libraries have highly consistent bulk statistics. More than 7 Gb of data from each library were obtained after filtering to remove low-quality reads and adapter sequences. The libraries yielded an average of 93.66% clean reads, and more than 93% of the total reads were mapped to the chicken reference genome in every library. The Q30 value was over 91% for every sample. These results demonstrated that the sequencing data from each library were of high quality. We used DESeq2 to screen for key candidate genes involved in ovary development and identified 415 differentially expressed genes between the H and L egg-laying groups (Figure 1A; Supplementary Table S4), of which 226 were upregulated and 189 were downregulated in the H group (Figure 1B). The log2 fold change values ranged from −7.08 to 8.87.

### Characterization of DEGs Using GO and KEGG Pathway Analyses

**FIGURE 2:**
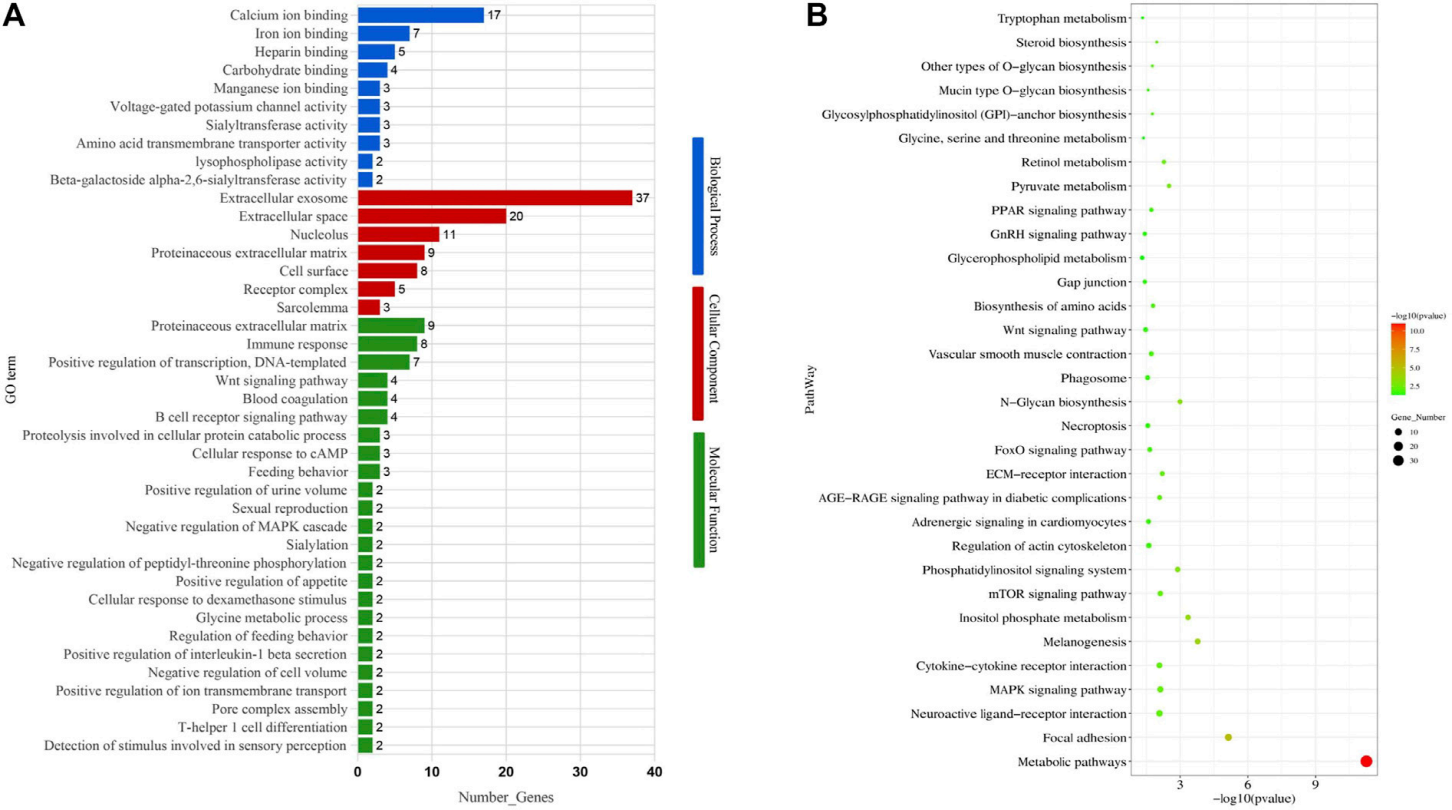
Gene Ontology (GO) terms and KEGG pathways enriched of differentially expressed genes. **(A)** GO terms. **(B)** KEGG pathways.

To identify the potential functional roles for the DEGs, we used GOseq to perform GO enrichment analysis. Within the category “biological process”, the most abundant GO terms were “extracellular exosome,” “positive regulation of protein kinase B signaling,” and “proteolysis involved in the cellular protein catabolic process.” For the “cellular component” and “molecular function” categories, the GO terms included “integral component of plasma membrane,” “proteinaceous extracellular matrix,” and “extracellular exosome” (Figure 2A; Supplementary Table S5).

To further identify gene functions and interactions, we also subjected the DEGs to KEGG pathway analysis, resulting in the identification of 105 enriched pathways (*p* < 0.05). Out of these, 32 enriched pathways were related to follicular development, including the GnRH signaling pathway, MAPK signaling pathway, steroid biosynthesis, gap junction, and mTOR signaling pathway (Figure 2B; Table 1).

**TABLE 1:** Significantly enriched pathways with functions in follicle development.

Pathway ID	Pathway term	Target gene list
gga01100	Metabolic pathways	NADK, MSMO1, ASL2, PCK1, ST6GAL2, NSDHL, ST6GAL1, DHTKD1, SMPD1, PGAM1, PLA2G4A, CYP27A1, GCNT2, ACAT2, MGAT5B, BCO1, NDUFA12, GLO1, PLCB2, ISYNA1, CRYL1, CYP21A1, PIGK, MAN2A2, PIGS, PHGDH, GGT5, BLVRA, GALNT7, HPSE, NME6, INPP5D, GALNT10, PIP5K1B, CDS2, INPP5K, ELOVL6, CYP26A1, ATP6V1C2
gga04510	Focal adhesion	PGF, LAMA1, LAMA2, PDGFC, VAV3, PARVG, CAPN2, COL6A2, EGF, THBS4
gga04916	Melanogenesis	PLCB2, FZD4, GNAO1, WNT6, EDN1, WNT16
gga00562	Inositol phosphate metabolism	PIP5K1B, PLCB2, INPP5K, INPP5D, ISYNA1
gga00510	N-glycan biosynthesis	MGAT5B, ST6GAL2, MAN2A2, ST6GAL1
gga04070	Phosphatidylinositol signaling system	PIP5K1B, PLCB2, INPP5K, INPP5D, CDS2
gga00620	Pyruvate metabolism	GLO1, ACAT2, PCK1
gga00830	Retinol metabolism	PNPLA4, BCO1, CYP26A1
gga04512	ECM–receptor interaction	LAMA1, LAMA2, COL6A2, THBS4
gga04010	MAPK signaling pathway	PGF, TGFA, PDGFC, CSF1R, MAPK12, PLA2G4A, EGF
gga04150	mTOR signaling pathway	ATP6V1C2, SLC7A5, FZD4, WNT6, WNT16
gga04933	AGE-RAGE signaling pathway in diabetic complications	EDN1, PLCB2, PRKCZ, MAPK12
gga04080	Neuroactive ligand–receptor interaction	OPRM1, CCKAR, LEPR, GRIN2C, NPY2R, EDN1, MC5R, P2RX7
gga04060	Cytokine–cytokine receptor interaction	CCL4, CSF1R, IL18R1, LEPR, TNFRSF11B, CXCL14
gga00100	Steroid biosynthesis	MSMO1, NSDHL
gga01230	Biosynthesis of amino acids	ASL2, PHGDH, PGAM1
gga00514	Other types of O-glycan biosynthesis	ST6GAL2, ST6GAL1
gga00563	Glycosylphosphatidylinositol (GPI)-anchor biosynthesis	PIGS, PIGK
gga03320	PPAR signaling pathway	CYP27A1, PCK1, FABP3
gga04270	Vascular smooth muscle contraction	EDN1, PLCB2, PLA2G4A, RAMP2
gga04068	FoxO signaling pathway	MAPK12, PCK1, EGF, FBXO32
gga04810	Regulation of actin cytoskeleton	BRK1, PIP5K1B, VAV3, PDGFC, EGF
gga04261	Adrenergic signaling in cardiomyocytes	PLCB2, MAPK12, PPP2R2C, ATP1B1
gga00512	Mucin-type O-glycan biosynthesis	GALNT7, GALNT10
gga04217	Necroptosis	PLA2G4A, HMGB1, SMPD1, CAPN2
gga04145	Phagosome	ATP6V1C2, THBS4, MARCO, DMB2
gga04310	Wnt signaling pathway	WNT16, PLCB2, FZD4, WNT6
gga04540	Gap junction	PLCB2, PDGFC, EGF, DRD1
gga04912	GnRH signaling pathway	PLCB2, PLA2G4A, MAPK12
gga00260	Glycine, serine, and threonine metabolism	PHGDH, PGAM1
gga00380	Tryptophan metabolism	DHTKD1, ACAT2
gga00564	Glycerophospholipid metabolism	CDS2, LPGAT1, PLA2G4A

### DEGs Validation by RT-qPCR

**FIGURE 3:**
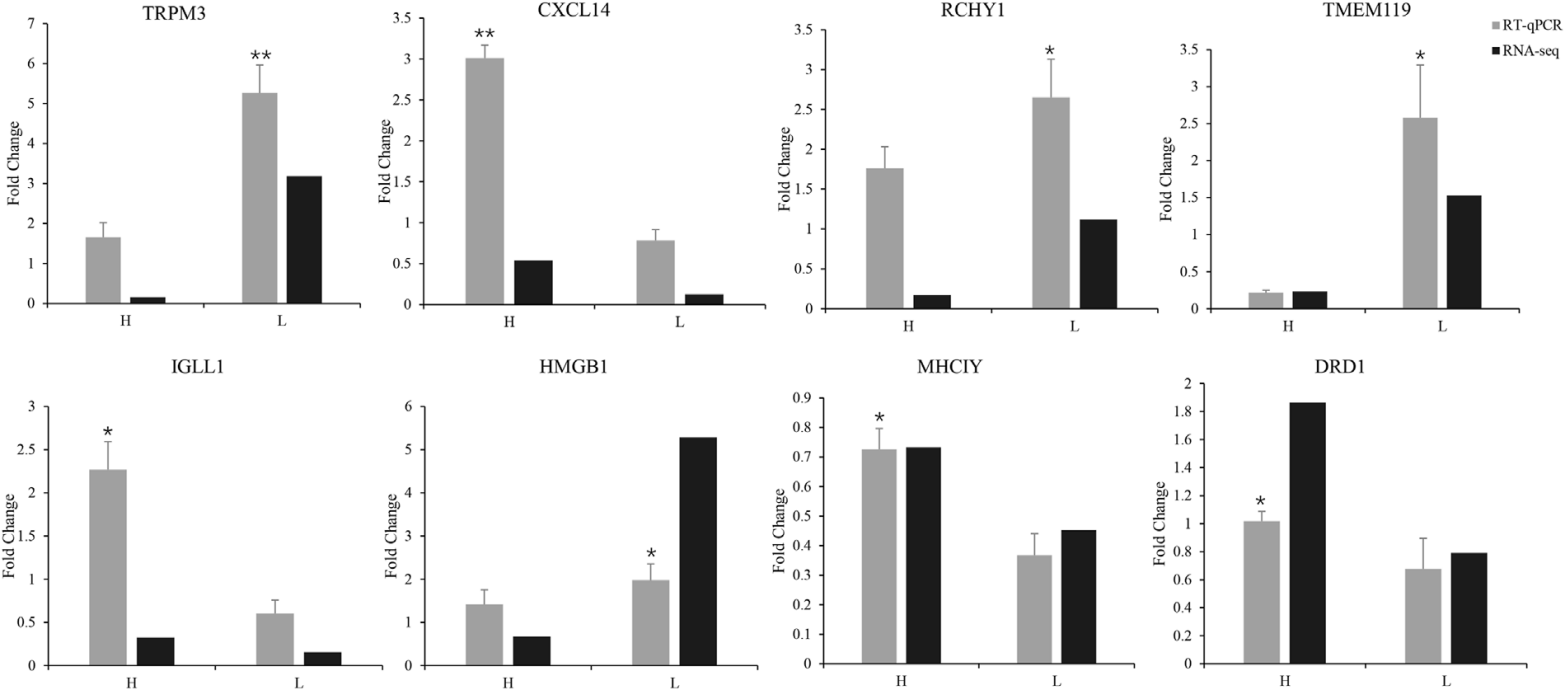
RT-qPCR validation of the expression of eight differentially expressed genes compared with mRNA transcriptome sequencing results (*p < 0.05, **p < 0.01).

To validate the expression levels of the DEGs, we performed RT-qPCR analysis on four upregulated and four downregulated genes. The upregulated genes were D (1) dopamine receptor protein (*DRD1*), MHC-like class I Y protein (*MHCIY*), C-X-C motif chemokine 14 (*CXCL14*), and immunoglobulin lambda-like polypeptide 1 (*IGLL1*). The downregulated genes were RING finger and CHY zinc finger domain-containing protein 1 (*RCHY1*), high- mobility group protein B1 (*HMGB1*), transmembrane protein 119 (*TMEM119*), and transient receptor potential cation channel subfamily M member 3 protein (*TRPM3*). The expression levels of these genes were significantly different between the H and L groups (shown in Figure 3, *p* < 0.05 or *p* < 0.01). The RT-qPCR results were consistent with our RNA-seq data and demonstrated that the DEGs identified by RNA-seq were reliable and accurate.

### Expression of miR-302a-3p and *DRD1* in SYFLs

**FIGURE 4:**
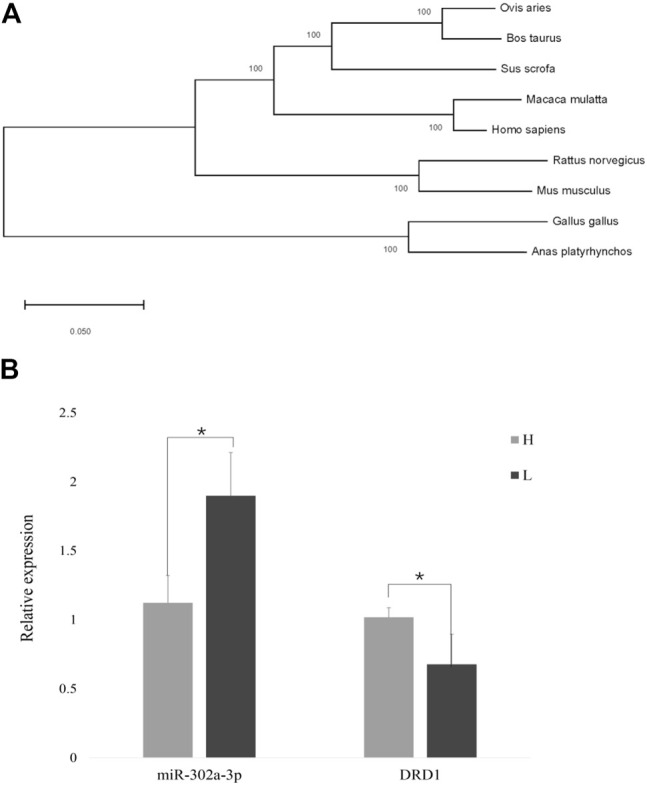
**(A)** Cluster plot showing *DRD1* conservation in various species. **(B)** Expression of *DRD1* and miR-302a-3p in small yellow follicles of high– and low–egg-yielding Taihang chickens (*p < 0.05).

**FIGURE 5:**
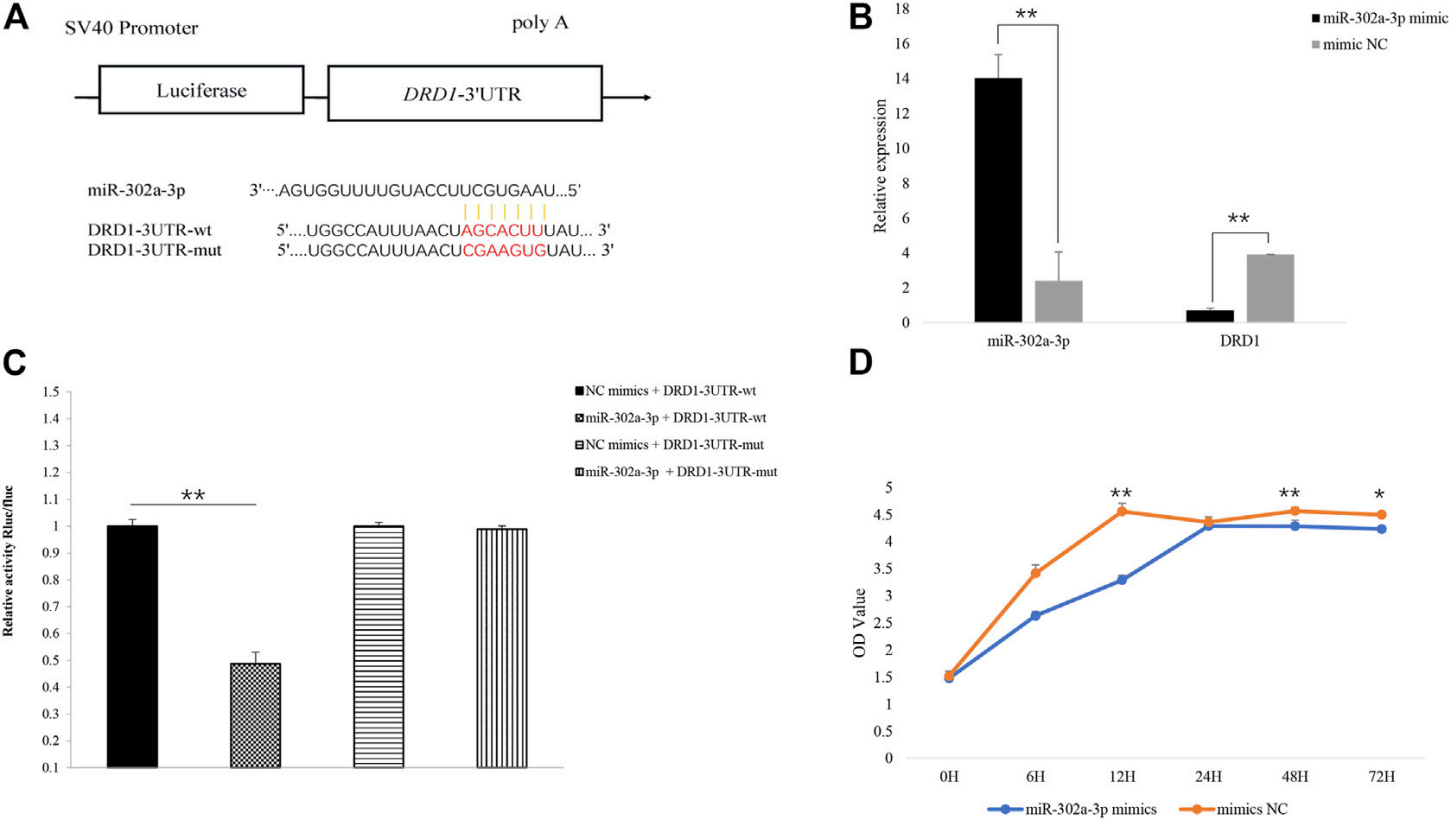
**(A)** Predicted binding site of miR-302a-3p on the 3′UTR of *DRD1* and vector construction. **(B)** Overexpression of miR-302a-3p inhibited the expression of *DRD1* in chicken granulosa cells. **(C)** Verification of miR-302a-3p binding to the 3′UTR of *DRD1* using dual-luciferase assay. **(D)** Overexpression of miR-302a-3p promoted chicken GC proliferation. *p < 0.05, **p < 0.01.

The gap junction channels are an important way for the GCs to pass nutrition to an oocyte (Starich et al., 2014). In the gap junction pathway, *DRD1* was significantly enriched. Cluster analysis showed that chicken *DRD1* is highly homologous with that of ducks and second with that of mice (Figure 4A). As can be seen from the relative expression of miR-302a-3p and *DRD1* in the H and L groups (Figure 4B), there is a negative correlation between the expression of miR-302a-3p and *DRD1*. This analysis indicated that *DRD1* may be a promising target for the genetic improvement of chicken egg production. A predicted binding site for miR-302a-3p was found in the *DRD1* 3′-UTR region (Figure 5A), and dual-luciferase reporter assays demonstrated that miR-302a-3p binds to the 3′UTR of *DRD1* (Figure 5C).

### Overexpression of miR-302a-3p Inhibits GC Proliferation by Targeting DRD1

To validate the function of miR-302a-3p, miR-302a-3p was overexpressed in chicken GCs by transfection. We found that *DRD1* expression was significantly inhibited in the miR-302a-3p overexpressing GCs (*p* < 0.01) (Figure 5B). Overexpression of miR-302a-3p inhibited cell proliferation, which was measured by CCK-8 (Figure 5D). These data indicated that miR-302a-3p inhibits the proliferation of GCs.

## Discussion

Egg production is a complex trait determined by genetics and numerous other factors (Zhao et al., 2019). In recent years, the understanding of egg production mechanisms has been greatly expanded as RNA-seq has revealed numerous genes that appear to be related to egg production, for example, *DRD1*, *MC5R*, *PCK1*, *CTSA*, and *TGFBR3* (Mishra et al., 2020). As we report here, a proportion of the DEGs we identified between high-yielding and low-yielding egg layers also play an important role in follicle development.

Several pathways involved in egg production were significantly enriched, including the GnRH signaling pathway (gga04912), oocyte meiosis (gga04114), progesterone-mediated oocyte maturation (gga04914), and the calcium signaling pathway (gga04020). The GnRH signaling pathway is an important signal transduction pathway for fertility in animals. GnRH stimulates the synthesis and release of gonadotropins and induces estrogen production and ovulation (Park et al., 2018). The calcium signaling pathway drives key events surrounding fertilization and activation of development in all species studied to date. Elevated intracellular Ca^2+^ concentrations are associated with processes that release unfertilized eggs from meiosis and trigger embryonic development (Miao and Williams, 2012). In egg production and reproduction pathways, the epidermal growth factor (EGF) is also involved in the regulation of the actin cytoskeleton, while PGF functions in corpus luteum proliferation and cell death (Ding et al., 2020).

*DRD1* is one of seven highly homologous cross-domain members of the G-protein–coupled receptor family (Wang et al., 2012). Dopamine promotes prolactin (PRL) secretion *via* DRD1 at the hypothalamic level, while DRD2 inhibits the secretion of PRL (Youngren et al., 1998; Al et al., 2003). In a study of the production traits of the Hungarian yellow chicken, *DRD1* was associated with egg strength and body weight (Tempfli et al., 2015). The expression of *DRD1* undoubtedly increases PRL secretion, resulting in an increase in brooding behavior frequency. In a transcriptomic analysis of atrophic ovaries, brooding hens had significantly higher PRL secretion levels than normal laying hens (Liu et al., 2018). Hens stop laying during brooding (Jiang et al., 2010), resulting in ovarian atrophy and an increase in white follicles, which slows development making it difficult for SYFLs to develop (Liu et al., 2009). The laying performance depends to some extent on ovary development, and *DRD1* may be related to brooding behavior (Xu et al., 2010). In this study, a homology analysis showed that *DRD1* was highly conserved in various species, which indicates that the function of *DRD1* is similar in these species. Our RNA-seq data and RT-qPCR analysis showed that the expression of *DRD1* was significantly higher in SYFs of high–egg-yielding chickens than in low–egg-yielding chickens. That *DRD1* plays a role in egg production and follicle development was consistent with previous studies.

Our results showed that *DRD1* is a target of gga-miR-302a-3p. miRNAs regulate gene expression by translational inhibition or by binding to the 3′ UTR of targeted genes. They regulate a variety of processes involved in development, organ formation, cell proliferation, and apoptosis (Kang et al., 2013). miRNAs participate in the production of steroids and can regulate human ovary GC proliferation and apoptosis (Sirotkin et la., 2009; Sirotkin et al., 2010). Eleven miRNAs have been identified in the chicken ovary that play regulatory roles in ovary and follicle development (Wu et al., 2017). miR-503, miR-672, and miR-465 are expressed in the mouse ovary and play an important role in the regulation of follicle development (Ahn et al., 2010). A reduction of miR-302 expression may be involved in reduced development in early embryogenesis (Parvin et al., 2019). Therefore, the role of miRNAs in follicle development cannot be ignored.

## Conclusion

In summary, RNA-seq analysis indicates that *DRD1* is involved in egg production. Functional assays showed that miR-302a-3p affects follicle differentiation by targeting *DRD1*. These results provide a foundation for improving chicken egg production, and further, our understanding of the development of chicken follicles.

## Data Availability

The original contributions presented in the study are publicly available. This data can be found here: NCBI, PRJNA796736, https://www.ncbi.nlm.nih.gov/bioproject/PRJNA796736/.

## Abbreviations

CCK-8, Cell Counting Kit-8; DEGs, differentially expressed genes; DRD1, dopamine receptor; FPKM, Fragments per Kilobase Millions; GCs, granulosa cells; GO, Gene Ontology; H, high egg production group; KEGG, Kyoto Encyclopedia of Genes and Genomes; L, low egg production group; MUT, mutant type; NC, negative control; PRL, prolactin; SYFLs, small yellow follicles; WT, wild type.
