# Supplementary material for: Transcriptome Analysis Reveals miR-302a-3p Affects Granulosa Cell Proliferation by Targeting DRD1 in Chickens
Source: Front Genet. 2022 Mar 30;13:832762. doi: 10.3389/fgene.2022.832762 (PMC9006144; doi:10.3389/fgene.2022.832762)
Supplement: Supplementary file 1 [file Table1.DOCX]

**Supplementary Table S1** Primer sequences used for RT-qPCR analysis

| Gene name | Sequence 5’-3’ | Tm(℃) |
| --- | --- | --- |
| MHCIY | 5' AGGAGAGATCACCCACCTGC 3' | 60 |
|  | 5' CGGCTCCCATGAGAAGAGAC 3' |  |
| TRPM3 | 5' TCTGATTGGCAAAGATGTGG 3' | 60.5 |
|  | 5' CCTTGACCAATTCGCGTAT 3' |  |
| CXCL14 | 5' TGATTATCGTGACCCTGTGG 3' | 59.2 |
|  | 5' CTTCATAAACCCTGCCCTTC 3' |  |
| RCHY1 | 5' GCTTGGAGGACATTCACACG 3' | 58.9 |
|  | 5' TCGTTACAAAGGATCTCCACC 3' |  |
| IGLL1 | 5' AACCCTGACCGTCCTAGGC 3' | 59 |
|  | 5' CCGTTGTGTGTGACCCTGC 3' |  |
| HMGB1 | 5' GATCCCAAGAAGCCGAGAG 3' | 58.5 |
|  | 5' GTCTTCCATCGTTCTGAGCAT 3' |  |
| INPP5K | 5' CGCTGTGAGAGAACTGAGGC 3' | 60 |
|  | 5' CGGATGGAGGAGAGCTTGAT 3' |  |
| TMEM119 | 5' GATGGGTTGGTGGCTTTC 3' | 59 |
|  | 5' TTTGGGGAAGGAGGATGG 3' |  |
| DRD1 | 5' TGTGCTCAACAGCCTCCAT 3' | 59.8 |
|  | 5' CGTGGTTGTAGCCTTGTGC 3' |  |
| GAPDH | 5' GCCCAGAACATCATCCCA 3' | 60 |
|  | 5' CGGCAGGTCAGGTCAACA 3' |  |
