# Supplementary material for: Transcriptome Analysis Reveals miR-302a-3p Affects Granulosa Cell Proliferation by Targeting DRD1 in Chickens
Source: Front Genet. 2022 Mar 30;13:832762. doi: 10.3389/fgene.2022.832762 (PMC9006144; doi:10.3389/fgene.2022.832762)
Supplement: Supplementary file 2 [file Table2.DOCX]

**Supplementary Table S2**: The sequence of DRD1-wt and DRD1-mut used for Dual Luciferase Reporter Assays

DRD1-3’UTR-wt:
GTAAAGGTAGGTGCATGCCTTCATAAATTATTTCTAAAACATTAATTGAGGCTTACAGTAGGAGTGAGAAATTTTTTTCCAGAATTGAGAGATGTTTTGTTGATATTGGTTCTATTTATTTATTGTATATATGGATATTTTTAATTTATGATATAATAAATATATATTTATCATATTTAATAGGATAAATTAATGAGTTTTATCCAAGACCTTACAACCACATTTCTGGCCATTTAACTAGCACTTTATAAGCCAATGAAGCAAACACACAGACTCTGTGAGATTCTAAATGTTCATGTGTAACTTCTAGAAACACAGCAGAAACTGATAGATAAGGGAATAAAGTTGAAATGATTCCTTAAAATTCATGGACACAGATAAATGCAAGGTGAGAATTGACAAATGCTATAAATGCTTTCTTTTTCTGAAAAGATTTTGAAAAATTTAAAAAAGTATAGCTACTACTGTGTTCAAAACGTTTTA

DRD1-3’UTR-mut:
GTAAAGGTAGGTGCATGCCTTCATAAATTATTTCTAAAACATTAATTGAGGCTTACAGTAGGAGTGAGAAATTTTTTTCCAGAATTGAGAGATGTTTTGTTGATATTGGTTCTATTTATTTATTGTATATATGGATATTTTTAATTTATGATATAATAAATATATATTTATCATATTTAATAGGATAAATTAATGAGTTTTATCCAAGACCTTACAACCACATTTCTGGCCATTTAACTcGaAgTgTATAAGCCAATGAAGCAAACACACAGACTCTGTGAGATTCTAAATGTTCATGTGTAACTTCTAGAAACACAGCAGAAACTGATAGATAAGGGAATAAAGTTGAAATGATTCCTTAAAATTCATGGACACAGATAAATGCAAGGTGAGAATTGACAAATGCTATAAATGCTTTCTTTTTCTGAAAAGATTTTGAAAAATTTAAAAAAGTATAGCTACTACTGTGTTCAAAACGTTTTA
