# Supplementary material for: Transcriptome Analysis Reveals miR-302a-3p Affects Granulosa Cell Proliferation by Targeting DRD1 in Chickens
Source: Front Genet. 2022 Mar 30;13:832762. doi: 10.3389/fgene.2022.832762 (PMC9006144; doi:10.3389/fgene.2022.832762)
Supplement: Supplementary file 4 [file Table3.DOCX]

**Supplementary Table S3** RNA sequencing statistics for 6 samples

| Sample | L-1 | L-2 | L-3 | H-1 | H-2 | H-3 |
| --- | --- | --- | --- | --- | --- | --- |
| Raw Reads | 46,500,676 | 47,700,656 | 47,471,974 | 44,044,756 | 43,492,010 | 42,159,198 |
| Raw Bases | 6,975,101,400 | 7,155,098,400 | 7,120,796,100 | 6,606,713,400 | 6,523,801,500 | 6,323,879,700 |
| Clean Reads | 44,616,078 | 44,810,512 | 43,842,000 | 41,887,406 | 39,808,264 | 39,258,668 |
| Clean Reads Rate(%) | 95.95 | 93.94 | 92.35 | 95.1 | 91.53 | 93.12 |
| Clean Bases | 6,692,411,700 | 6,721,576,800 | 6,576,300,000 | 6,283,110,900 | 5,971,239,600 | 5,888,800,200 |
| Low-quality Reads | 319,956 | 303,930 | 369,212 | 292,304 | 271,936 | 333,884 |
| Low-quality Reads Rate(%) | 0.69 | 0.64 | 0.78 | 0.66 | 0.62 | 0.79 |
| Ns Reads | 2,822 | 2,836 | 2,722 | 2,708 | 2,566 | 2,622 |
| Ns Reads Rate(%) | 0.01 | 0.01 | 0.01 | 0.01 | 0.01 | 0.01 |
| Adapter Polluted Reads | 1,561,820 | 2,583,378 | 3,258,040 | 1,862,338 | 3,409,244 | 2,564,024 |
| Adapter Polluted Reads Rate(%) | 3.36 | 5.42 | 6.86 | 4.23 | 7.84 | 6.08 |
| Raw Q30 Bases Rate(%) | 91.69 | 91.97 | 90.82 | 91.86 | 91.6 | 91.22 |
| Clean Q30 Bases Rate(%) | 91.99 | 92.25 | 91.09 | 92.15 | 91.85 | 91.56 |
